# Dosing of plant-derived extracellular vesicles in traditional Chinese medicine: current status, root causes, and standardization pathways

**DOI:** 10.3389/fphar.2026.1876668

**Published:** 2026-07-08

**Authors:** Mingxin Guo

**Affiliations:** Department of Pharmacy, The Affiliated Yixing Hospital of Jiangsu University, Yixing, Jiangsu, China

**Keywords:** dosage, extracellular vesicles, nanomedicine, standardization, traditional Chinese medicine, vesicle yield

## Abstract

Extracellular vesicles derived from Traditional Chinese Medicine represent a growing research Frontier. However, no consensus exists regarding the definition, quantification, and reporting of administered doses. This methodological issue continues to compromise study reproducibility. This article draws on a comprehensive analysis of recent and foundational literature on TCM-derived extracellular vesicles. Analysis shows marked differences in measurement units, reporting formats, and magnitude ranges for both *in vitro* and *in vivo* dosing. Vesicle yields also vary considerably depending on plant species, extraction method, and harvest season. The inconsistency of dose reporting and the incomparability of yield data together impede clinical translation. Further examination identifies three root causes: inherent fluctuations from batch-to-batch variability of herbal materials, the prolonged absence of quantitative standards for plant vesicles, and - as a core conceptual finding - the divergent dosing logic arising from the dual identity of vesicles as both pharmacologically active entities and nanocarriers. Based on these findings, this article proposes several standardization pathways. These include establishing multi-dimensional reporting norms for dose and yield, constructing species-specific efficacy equivalent systems, and bridging the metrological gap between traditional herbal dosage and modern nanomedicine dosing.

## 1 Introduction

Extracellular vesicles (EVs) derived from Traditional Chinese Medicine (TCM), often termed plant-derived nanovesicles (PDNVs) —a term used interchangeably in the literature with exosome-like nanovesicles (ELNs) or plant-derived extracellular vesicles (PDEVs); hereinafter uniformly referred to as PDNVs, are a prominent research focus. These phospholipid bilayer-enclosed nanoparticles carry nucleic acids, proteins, lipids, and secondary metabolites from their source plants. They demonstrate remarkable therapeutic effects in a wide array of preclinical models. *Lonicera japonica* Thunb. [Caprifoliaceae; Lonicerae japonicae flos]-derived nanovesicles alleviate inflammatory bowel disease by modulating the gut microbiota-immune axis (Wang Y. et al., 2025). *Artemisia argyi* H. Lév. & Vaniot [Asteraceae; Artemisiae argyi folium]-derived exosome-like nanovesicles mitigate acute liver failure via the TLR4/NLRP3/Nrf2 axis (Zheng et al., 2026). *Panax notoginseng* (Burkill) F.H.Chen ex C. Chow [Araliaceae; Notoginseng radix et rhizoma]-derived vesicles deliver miRNA across kingdoms to restore neuronal function after ischemic stroke (Yu et al., 2026). *Polygonum multiflorum* Thunb. [Polygonaceae; Polygoni multiflori radix]-derived exosome-like nanoparticles promote hair growth by modulating the human androgen receptor pathway via plant miRNAs (Li S. et al., 2025). The publication rate of related papers in leading journals such as *Phytomedicine*, *Pharmacological Research*, and *Journal of Nanobiotechnology* is increasing exponentially.

Despite these impressive findings, a pressing methodological issue demands collective attention. One crucial clarification concerns the ongoing debate regarding cross-kingdom delivery of plant miRNAs. Several studies have demonstrated that exosome-like nanoparticles from Panax notoginseng and Polygoni Multiflori Radix can deliver miRNAs across kingdoms to exert therapeutic effects (Yu et al., 2026; Li S. et al., 2025). However, contradictory evidence must be acknowledged. Snow et al. (2013) showed that plant miRNAs (MIR156a, MIR159a, and MIR169a) present in mouse diets failed to achieve detectable bioavailability in serum or liver after oral administration, challenging the feasibility of cross-kingdom RNA delivery (Snow et al., 2013). Similarly, Dickinson et al. reported that orally administered plant miRNAs did not accumulate in mammalian tissues (Dickinson et al., 2013). This ongoing debate has direct implications for dose logic: if cross-kingdom miRNA delivery remains controversial, then a substantial portion of the proposed therapeutic mechanism may need to be reconsidered, and dose standardization must be anchored on alternative pharmacodynamic readouts rather than assuming functional miRNA transfer.

The metrics and magnitude of administered doses vary so widely that they defy a unified scientific rationale. Crucially, the dosing problem is inseparable from vesicle yield data. If laboratories cannot agree on how many vesicles are extracted from a given amount of raw herb, any discussion of dosage lacks a fundamental reference point. A 2025 systematic review by *Zhang* et al. in *Phytomedicine* explicitly noted prominent challenges. These include diverse sources, complex compositions, unclear mechanisms, and a lack of uniform standards for isolation and characterization (Zhang et al., 2025). An earlier systematic analysis by Gupta et al. (2021) in *Advanced Drug Delivery Reviews* also highlighted significant disparities in effective dosing strategies across the broader EV therapeutics field. They noted that most studies do not reference published pharmacokinetic or biodistribution data when selecting experimental doses (Dhanu et al., 2021). In the specialized area of TCM-derived vesicles, plant material variability adds another layer of complexity to an already challenging field. The demand for methodological rigor is therefore urgent.

This article provides a systematic analysis of dosing issues in TCM-derived vesicle research. First, we use a comprehensive literature analysis to map the heterogeneity in dose reporting and vesicle yields from recent representative studies. Second, we explore the deeper roots of this heterogeneity. Third, we evaluate recent standardization initiatives and their limitations. Finally, we propose pathways toward a unified dosing framework. Our goal is to provide a theoretical reference for the field’s transition from exploratory studies to a more rigorous and quantitatively standardized era.

## 2 Current status of dose reporting

We searched databases including PubMed, CNKI, and Web of Science to identify relevant literature on dose reporting and vesicle yield in TCM-derived extracellular vesicle research. Representative studies, encompassing both recent and foundational work, were selected for analysis. All plant names in this review have been taxonomically validated using the Medicinal Plant Names Services (MPNS, http://mpns.kew.org) and Plants of the World Online (http://www.plantsoftheworldonline.org) databases. Nomenclature follows the International Code of Nomenclature for algae, fungi, and plants (ICN). Table 1 illustrates the significant heterogeneity in dosing units, reporting formats, and magnitude ranges, even among recent high-quality publications. For each study, Table 1 also reports the extraction method used and provides a purity notation where available, allowing readers to directly link dose metrics to sample preparation.

**TABLE 1:** Administered doses in representative studies of TCM-derived extracellular vesicles.

Plant source (Authority, family; pharmacopoeia name)	Disease model	Administration route	*In* *vivo* dose	*In* *vitro* dose	Extraction method (purity grade)	Yield	References
*Lonicera japonica* Thunb. [Caprifoliaceae; lonicerae japonicae flos]	Cisplatin-induced AKI (mice)	Intraperitoneal	30 mg/kg/d, 5 days	50–200 μg/mL (HK-2 cells); 100 μg/mL optimal	Ultracentrifugation (purity not reported)	—	Gong et al. (2025)
*Artemisia argyi* H.Lév. and Vaniot [Asteraceae; Artemisiae argyi folium]	DSS-induced colitis (mice)	Oral	25 μg/kg and 50 μg/kg	—	Differential centrifugation (purity not reported)	—	Li et al. (2025b)
*Artemisia argyi* H.Lév. and Vaniot [Asteraceae; Artemisiae argyi folium]	LPS/D-GalN-induced ALF (mice)	Oral	Not specified	—	Differential ultracentrifugation (purity not reported)	—	Zheng et al. (2026)
*Panax notoginseng* (Burkill) F.H.Chen ex C.Chow [Araliaceae; notoginseng radix et rhizoma]	Cerebral ischemia-reperfusion (mice)	Tail vein injection	1.0 mg/kg	10–50 μg/mL (HT22/N2a); 20 μg/mL optimal	Ultracentrifugation (purity not reported)	—	Yu et al. (2026)
*Polygonum multiflorum* Thunb. [Polygonaceae; Polygoni multiflori radix]	Androgenetic Alopecia (mice)	Topical (Dorsal Skin)	Not specified	25–100 μg/mL (HHDPC); 50 μg/mL optimal	Differential ultracentrifugation (purity not reported)	2.22 × 10^9^ particles/g raw herb	Li S. et al. (2025)
*Panax ginseng* C.A.Mey. [Araliaceae; ginseng radix et rhizoma]	Non-small cell lung cancer (mice)	Intravenous (+anti-PD-l1)	10 μg/mouse (+anti-PD-l1 8 μg)	10 μg/mL (A549/H1299)	Ultracentrifugation (purity not reported)	—	Zhu et al. (2025)
*Atractylodes macrocephala* Koidz. [Asteraceae; Atractylodis macrocephalae rhizoma]	DSS-induced colitis (mice)	Oral	2 mg/kg (protein basis)	Concentration-dependent	Differential centrifugation (purity not reported)	—	Tan et al. (2025)
*Portulaca oleracea* L. [Portulacaceae; Portulacae herba]	Diabetic Wound (mice)	Topical	—	Concentration-dependent	Ultracentrifugation (fresh material, higher bioactivity)	—	Song et al. (2025)
*Lycium ruthenicum* Murray [Solanaceae; —]	Aβ-induced HT22 cell model	Medium Supplement	—	Not specified	PEG precipitation (low purity)	4.24 g/kg raw herb	Zhang et al. (2024)
*Zingiber officinale* Roscoe [Zingiberaceae; Zingiberis rhizoma recens]	—	—	—	—	Precipitation (low purity)	9.2 mg lyophilized mass/g raw herb	Li et al. (2025b)
*Zingiber officinal*e Roscoe [Zingiberaceae; Zingiberis rhizoma recens]	—	—	—	—	Ultracentrifugation (higher purity)	1.63 g protein/kg raw herb	Song et al. (2025)
*Allium sativum* L. [Amaryllidaceae; Allii sativi bulbus]	—	—	—	—	Precipitation (low purity)	6.0 mg lyophilized mass/g raw herb	Li et al. (2025b)
*Curcuma longa* L. [Zingiberaceae; Curcumae longae rhizoma]	—	—	—	—	Precipitation (low purity)	8.6 mg lyophilized mass/g raw herb	Li et al. (2025b)
*Millettia specios*a Champ. [Fabaceae; Millettiae speciosae radix]	—	—	—	—	Patented method (qualitative)	High yield, rapid isolation (qualitative)	Wang Q. et al. (2025b)
*Artemisia absinthium* L. [Asteraceae; Absinthii herba]	—	—	—	—	Differential centrifugation (relative comparison)	Significantly higher than others (relative comparison)	Affiliated Hospital of Guangdong Medical University (2026)

“-” indicates the item was not applicable or dose data was not specified in the literature.

Regarding *in vivo* doses, the metrics used vary considerably. For instance, Gong et al. used a dose of 30 mg/kg/d (based on vesicle mass) for honeysuckle vesicles in a cisplatin-induced acute kidney injury mouse model. This was administered via intraperitoneal injection for five consecutive days (Gong et al., 2025). In contrast, studies on *Atractylodes macrocephala* Koidz. [Asteraceae; Atractylodis macrocephalae rhizoma] vesicles employed a different metric. The authors isolated vesicles with a protein concentration of 8,330 μg/mL. The *in vivo* dose was reported as 2 mg/kg based strictly on protein assay (Tan et al., 2025). It is important to note that this protein-based dosing implicitly assumes a constant protein-to-lipid ratio across extraction batches—an assumption that is highly questionable given the substantial batch-to-batch variability in vesicle purity and composition. In a study of *Panax ginseng* C.A.Mey. [Araliaceae; Ginseng radix et rhizoma]-derived vesicles for non-small cell lung cancer, the *in vivo* dose was stated as 10 μg per mouse, administered intravenously in combination with 8 μg of anti-PD-L1 monoclonal antibody every other day for 20 days (Zhu et al., 2025). This combination therapy context is critical because the presence of an immune checkpoint inhibitor fundamentally alters the baseline dosing strategy, shifting the vesicle’s role from a standalone therapeutic to a synergistic adjuvant. For *P. notoginseng* vesicles treating cerebral ischemia-reperfusion injury, the *in vivo* dose was 1.0 mg/kg administered via tail vein injection (Yu et al., 2026).

A striking observation emerges when comparing doses across studies using similar disease models. Both Artemisia argyi EVs (25 μg/kg, oral) and honeysuckle EVs (30 mg/kg, intraperitoneal) have been tested in mouse colitis or injury models (Zheng et al., 2026; Gong et al., 2025). The ∼1000-fold difference in reported doses could be attributed to three competing factors: (i) genuine pharmacological potency differences, (ii) incompatible dose metrics (protein content vs. total vesicle mass), or (iii) differing routes of administration (oral vs. intraperitoneal). However, a more fundamental confounder is that oral administration targets the gut microbiota locally, whereas intraperitoneal injection achieves systemic bioavailability; these two routes operate on entirely different dose-response curves and pharmacokinetic paradigms. The magnitude of difference—three orders of magnitude—strongly suggests that incompatible dose metrics and reporting inconsistencies, rather than true pharmacological potency, account for most of this variation. Furthermore, the two studies employed different disease models and different administration routes, complicating direct comparisons. This example underscores the urgent need for standardized dose metrics that enable cross-study comparability.

Reporting of *in vitro* doses is similarly non-uniform. Gong et al. tested concentrations ranging from 50 to 200 μg/mL for honeysuckle vesicles. They identified 100 μg/mL as the optimal therapeutic concentration (Gong et al., 2025). The ginseng vesicle study used an *in vitro* concentration of 10 μg/mL (Zhu et al., 2025). In the study of *P. notoginseng* vesicles for ischemia-reperfusion injury, researchers tested 10–50 μg/mL. They determined 20 μg/mL as optimal (Yu et al., 2026). Notably, the same *P. notoginseng* study reported a particle concentration of approximately 1 × 10^12^ particles/mL in the stock preparation, providing a crucial link between mass-based dosing discussions and particle count realities (Yu et al., 2026). For *Polygoni Multiflori Radix* vesicles promoting hair growth, *in vitro* experiments used 25–100 μg/mL. The optimal concentration was 50 μg/mL (Li S. et al., 2025).

However, studies providing quantitative dose data represent a minority. Many recent publications omitted specific dosage information entirely. Examples include studies on intranasal *Ganoderma lucidum* (Curtis) P. Karst. [Ganodermataceae; Ganoderma lucidum] vesicles for Alzheimer’s disease (Mi et al., 2025), oral *Pueraria lobata* (Willd.) Ohwi [Fabaceae; Puerariae lobatae radix] vesicles for rheumatoid arthritis (Han et al., 2025), *P. lobata* vesicles for non-alcoholic steatohepatitis (Ma et al., 2026), topical *P. notoginseng* vesicles for psoriasis (Chen XY. et al., 2026), and *Trichosanthes kirilowii* Maxim. [Cucurbitaceae; Trichosanthis fructus] vesicles for atherosclerosis (Chen YL. et al., 2026). The original texts or available abstracts of these papers provided no specific dose information. Notably, a substantial portion of the aforementioned studies were published in high-quality journals recognized in this field. These include *Phytomedicine*, *Journal of Nanobiotechnology*, and *Pharmacological Research*. This indicates that dose reporting heterogeneity is not confined to lower-quality research. It represents a pervasive methodological challenge facing the entire field.

## 3 Interrelation of vesicle yield and dose standardization

A key reason for the slow progress in dose standardization is the lack of comparable yield data across studies. If there is no consensus on how many vesicles are obtained from a specific mass of raw herb, dose discussions lack a fundamental anchor. Yield data are often missing or reported using disparate metrics. This prevents researchers from calibrating doses based on crude herb equivalents. Table 1 summarizes representative studies that explicitly reported vesicle yields for TCM materials.

As shown in Table 1, yield reporting methods are equally chaotic. First, measurement units are far from uniform. Some studies report particle count per Gram of raw herb. Others report protein mass per kilogram of raw herb or lyophilized vesicle mass per Gram of raw herb. A few provide only final particle concentration per milliliter. This makes it impossible to trace back to the input material.

Second, a fundamental physical metrological constraint must be recognized: vesicle mass scales with the cube of the radius, whereas particle count scales linearly with number of objects. Consequently, mass-based yield metrics are disproportionately skewed by a small number of co-isolated large microvesicles or cell debris particles, while particle-based metrics can be inflated by small non-vesicular aggregates. This volumetric scaling principle means that mass and particle count cannot be linearly converted across samples with different size distributions. When different laboratories use mass-based versus particle-based metrics, cross-study comparisons are severely compromised because the underlying physical dimensions are incommensurable (Dhanu et al., 2021).

Third, the heterogeneity in reported yields is frequently a symptom of purity heterogeneity rather than an actual reflection of vesicle abundance within the source plant. Precipitation-based methods co-isolate massive amounts of non-vesicular plant macromolecules, including polysaccharides, cell wall fragments, and soluble proteins such as Rubisco. These contaminants artificially inflate mass-based yields—sometimes by orders of magnitude—compared to density gradient ultracentrifugation, which removes most non-vesicular components (Gámez-Valero et al., 2016). For example, Table 1 shows that PEG precipitation of *Lycium ruthenicum* yielded 4.24 g of material per kg raw herb (Zhang et al., 2024), whereas differential ultracentrifugation of *Polygoni Multiflori Radix* yielded only 2.22 × 10^9^ particles per Gram (equivalent to micrograms of pure vesicle mass) (Li S. et al., 2025). The thousand-fold difference in mass-based yield between these two studies is largely explained by purity differences, not by genuine biological variation in vesicle abundance. Therefore, any discussion of dose standardization must first establish purity benchmarks; otherwise, comparing “yields” across methods compares apples to oranges.

Fourth, some patents and new technology reports use qualitative descriptions like “high yield” or “rapid separation” instead of specific quantitative metrics (Affiliated Hospital of Guangdong Medical University, 2026). This further reduces reproducibility. The dual absence of yield data and standardized reporting prevents readers from estimating the amount of raw herb corresponding to the vesicles used in an experiment. This severs any meaningful link between nanovesicle dosage and traditional crude herb dosage.

A promising methodological advance that could help standardize yields involves enzyme-assisted cell wall digestion. Wang et al. (2025) developed an optimized enzyme-based EVs extraction method, denoted Phyto-EVpure, which was validated across seventeen medicinal plants, including ginger, Morus alba leaves, and Isatis indigotica roots, using fresh, frozen, or dried starting materials (Wang Q. et al., 2025). The enzymatic pulping process improved EVs yield and purity over conventional grinding, particularly for dried vine samples that had previously been intractable (Wang Q. et al., 2025). From a dose standardization perspective, Phyto-EVpure offers several advantages: first, it reduces yield variability arising from differences in mechanical disruption efficiency; second, it enables consistent vesicle recovery from dried or processed herbal materials—a critical consideration given that TCM prescriptions often use dried, processed, or decocted herbs rather than fresh material; third, the method specifically addresses a major source of yield heterogeneity in Table 1—namely, the variation arising from extraction protocols. We suggest that future studies evaluating or comparing yields adopt this enzymatic approach as a reference baseline, which would greatly facilitate cross-laboratory comparability.

Before discussing enzymatic advances, however, it is essential to confront a conceptual pitfall: the notion of back-calculating a “crude herb equivalent” dose. The extraction efficiency of PDNVs—whether measured by particle count, protein mass, or lipid content—varies dramatically with extraction scale, method, and source material quality. A benchtop ultracentrifugation protocol yielding 10^9^ particles per Gram of fresh ginger cannot be assumed to scale linearly to pilot-scale manufacturing using dried ginger from a different harvest. Thus, any proposed “crude herb equivalent” must be rigorously defined as the mass of raw herb required to produce a given quantity of PDNVs under a specified, standardized extraction protocol. Extrapolation across protocols or scales requires empirical validation of extraction efficiency scaling factors. The field must avoid the naive assumption that a vesicle dose can be universally equated to a fixed herb mass.

Yield uncertainty also stems from biological variation in the source material. Evidence suggests that ginger harvested in July yields vesicles with better size distribution and stability compared to other months. Ambient temperature correlates negatively with vesicle size (Shokooh and Fereidoni, 2025). This means that two laboratories following identical extraction protocols might obtain significantly different yields and vesicle quality due solely to harvest timing. Another study comparing nanovesicle preparations from different plant sources confirmed species-specific variations in yield, size distribution, and antioxidant activity (Viršilė et al., 2022). Thus, vesicle yield is not merely a methodological issue. It is intertwined with intrinsic factors like plant species and growth conditions. This further compounds the difficulty of dose standardization.

## 4 Analysis of sources of dosage variation

The chaotic state of dose reporting and yield data is not due to individual researcher oversight. It arises from three intertwined root causes. These causes are inherent to the nature of TCM vesicle research.

### 4.1 Batch-to-batch variability of herbal materials

The metabolite composition of TCM is significantly influenced by geographic origin, harvest season, processing methods, and storage conditions. This is well-recognized (Ji et al., 2024). Traditionally, control is achieved through the designation of Dao-di herbs and standardized processing protocols. The introduction of EVs adds substantial complexity. Vesicles isolated from the same batch of herbs can vary in yield, size distribution, cargo content, and surface protein profile. These variations depend on the plant’s physiological state and the extraction workflow. Fresh versus dried material yields different results. Juice extraction differs from homogenization. Differential centrifugation yields different outcomes than density gradient ultracentrifugation. Any variation in these steps can alter the effective dose of the final vesicle product by an order of magnitude. A telling example comes from purslane research. A study comparing isolation methods found that vesicles obtained from fresh material via ultracentrifugation exhibited superior bioactivity. Multi-omics analysis confirmed higher levels of metabolites and proteins related to tissue regeneration in these fresh-source vesicles (Song et al., 2025). Consequently, one cannot assume that identical particle concentrations correspond to equivalent therapeutic effects across different laboratories or even across different batches prepared within the same lab.

An additional layer of complexity arises from the traditional processing of Chinese herbs, known as Paozhi. For example, raw Polygoni Multiflori Radix contains high levels of emodin and other anthraquinones that confer significant hepatotoxicity, whereas processed (cured) forms of the same herb exhibit hepatoprotective properties due to transformation of these metabolites during steaming. The PDNVs derived from raw versus processed Polygoni Multiflori Radix would carry dramatically different metabolite payloads and, consequently, different toxicity profiles and therapeutic windows. Therefore, standardized dosing cannot be defined solely in terms of particle count or protein mass; it must also incorporate strict toxicity bounds based on the specific processing state of the source herb. A vesicle preparation from processed herb might be safe at a dose that would be toxic if derived from raw herb. Current literature rarely specifies the Paozhi status of the starting material, representing a critical gap in dose reporting (Wang et al., 2023). This variability is visible in yield data. As mentioned earlier, ginger vesicle yield varies with harvest season (Shokooh and Fereidoni, 2025). Yield is a metric highly sensitive to the state of the source material. Since yield consistency cannot be achieved across laboratories, dose standardization remains elusive.

### 4.2 Absence of quantitative standards for plant vesicles

Mammalian EVs research has long benefited from the evolving MISEV guidelines. First published in 2014 and revised in 2018, the latest Minimal Information for Studies of Extracellular Vesicles 2023 (MISEV 2023) guidelines represent a significant update from the 2014 and 2018 versions, with expanded discussions on non-mammalian EVs, including plant-derived vesicles (Welsh et al., 2024). However, while MISEV states its principles apply to non-mammalian studies, specific technical recommendations, especially marker selection and validation, rely heavily on mammalian systems. Research indicates that directly applying MISEV guidelines to plant-microbe interaction contexts presents unique challenges (Rodríguez de Lope et al., 2025). Plant vesicles lack classic tetraspanin markers like CD9, CD63, and CD81 widely used in mammalian EVs research. Importantly, the MISEV2023 framework explicitly acknowledges ongoing efforts to validate plant-specific EV markers. These include fasciclin-like arabinogalactan proteins, germin-like proteins, and syntaxins such as PEN1 (PENETRATION1), which have been shown to enrich in plant extracellular vesicles. Incorporating these emerging biomarkers will be essential for establishing a uniquely plant-specific metrological framework that does not simply borrow mammalian standards. Cai et al. (2021) emphasized the urgent need for rigorous standards in plant EV research, particularly regarding isolation and characterization protocols (Cai et al., 2021). A proteomic analysis of Arabidopsis and *Brassica oleracea* EVs found that while tetraspanin families exist in plants, their use as Plant Extracellular Vesicle markers requires careful evaluation of species-specificity and enrichment. In contrast, families like fasciclin-like arabinogalactan proteins and germin-like proteins exhibit clearer plant-specific potential (Ha et al., 2025).

Nanoparticle tracking analysis (NTA) is a common technique for sizing and counting EVs. However, it has significant limitations. Conventional scatter-mode NTA cannot reliably distinguish vesicles from protein aggregates and other co-purified nanoparticles. This leads to discrepancies between particle counts and actual EV concentrations (Visnovitz et al., 2019). Moreover, NTA suffers from the swarm effect and is highly sensitive to the refractive index of the sample. Plant EV cargos contain variable concentrations of secondary metabolites and pigments that alter refractive index, making particle concentration measurements inconsistent across different plant species or even different batches of the same herb (Vestad et al., 2017). Not all NTA-counted particles possess equivalent biological activity. Some signals may derive from empty membrane vesicles, cell debris, or non-specific aggregates. More advanced technologies, such as nano-flow cytometry (nFCM) and digital dual CRISPR-Cas single vesicle evaluation systems, offer higher resolution and absolute quantification capabilities. These techniques can differentiate vesicle subpopulations based on surface markers and cargo, providing a much more rigorous technological pathway for absolute quantification than conventional NTA (Xu et al., 2025). Protein quantification also suffers from inconsistent standards. Studies indicate that relying solely on protein content or particle number to calibrate EV samples introduces errors. Lipid bilayer-based quantification, combined with protein or particle counts, is a more reliable approach (Zhang et al., 2016). The chaotic reporting of yields exemplifies this lack of quantitative standardization. At least four different measurement units were used across the listed studies. There is no accepted conversion pathway between them. If heterogeneous dose reporting is a surface-level problem, the incomparability of vesicle yields is a deeper structural issue. These two interconnected challenges make quantitative cross-study comparisons exceptionally difficult.

### 4.3 Overlapping identities: active ingredient and nanocarrier

A fundamental yet underappreciated reason for dosing confusion is the dual role played by TCM vesicles. On one hand, they are pharmacologically active substances. Encapsulated plant miRNAs, lipids, proteins, and secondary metabolites can directly modulate cell signaling, inhibit inflammation, scavenge reactive oxygen species, or promote tissue repair. On the other hand, they function as nanocarriers. The phospholipid bilayer structure allows for loading and delivery of exogenous drugs. Certain plant-derived vesicles exhibit natural tissue tropism. This property is being harnessed for targeted delivery system design (Cong et al., 2022).

These two roles imply two distinct dosing strategies. If vesicles are viewed as active ingredients, dose design should reference TCM frameworks. Doses should be based on crude herb equivalents and align with traditional empirical usage ranges. However, few studies investigate what mass of raw herb corresponds to the vesicle dose administered to a mouse. Because most studies do not report yields, calculating the crude herb equivalent is impossible. This approach remains unrealized. Conversely, if vesicles are viewed as nanocarriers, dose design should follow synthetic nanomedicine logic. Doses should be optimized based on pharmacokinetic (PK) parameters and biodistribution data. In the context of nanoparticles, key PK parameters include circulation half-life, systemic clearance, and biodistribution to the reticuloendothelial system. PDNVs administered intravenously must actively evade opsonization and phagocytosis by Kupffer cells, whereas orally administered PDNVs face an entirely different set of barriers—gastric acid degradation, enzymatic digestion, and mucus penetration—before reaching the gut epithelium. These fundamentally different PK bottlenecks mean that the same nominal dose can produce vastly different exposure at the target site depending on the route of administration (Alexis et al., 2008). The problem is that these two logics are used interchangeably in the literature. No effort is made to build a bridge between them. Researchers have pointed out that when plant-derived EVs are treated as nanomedicines, rigorous validation of their *in vivo* metabolism and safety profiles is required. Current research falls short in this regard (National Expert Committee on Research and Application of Chinese Herbal Vesicles, 2024). The review by Zhang et al. touches on this same dilemma. They note that while TCM-derived exosome-like nanovesicles show significant therapeutic potential, quality control and standardization issues persist. The review concludes with a call to prioritize the establishment of quality standards. This aligns closely with the core concern of this article (Zhang et al., 2025).

## 5 Current standardization efforts and limitations

It is encouraging that the TCM vesicle research community has begun to systematically address these challenges.

In 2024, the National Expert Committee on Research and Application of Chinese Herbal Medicine Vesicles published the “Expert Consensus on Research and Application of Chinese Herbal Medicine Vesicles (2023 Edition)” in the journal *Chinese Traditional and Herbal Drugs*. The consensus was developed using Delphi, consensus conference, and nominal group techniques. It addressed issues including research status, nomenclature, isolation methods, quality standards, and translational applications. Agreement was reached on 13 items. The consensus openly acknowledged prominent problems such as imprecise dosing and unstable efficacy. It identified the lack of standardization as the root cause. This consensus provides a valuable methodological reference for researchers in this field. An English version was published in *Chinese Herbal Medicines* in the same year, extending its international reach (Zhao et al., 2024).

A more significant milestone occurred in February 2026. The China Food and Drug Administration Enterprise Quality and Safety Promotion Association approved and published the group standard “Technical Requirements for Chinese Herbal Extracellular Vesicles Used as Drugs or Drug Carriers” (T/FDSA 0120-2026). Led by the Guangzhou National Laboratory, the standard took effect on 2 March 2026 (China Food and Drug Administration Enterprise Quality and Safety Promotion Association, 2026). This is the first technical standard globally specifically for Chinese herbal extracellular vesicles. It marks a transition from fragmented exploration to a phase with established standards. Nevertheless, we must maintain a clear-eyed assessment of what these efforts have achieved and what gaps remain. Both the 2023 Consensus and the 2026 Technical Standard focus primarily on technical requirements for isolation, purification, quality control, and characterization. They answer the question: *What constitutes a qualified vesicle product? They do not answer the question: How much of this qualified product should be administered?* More specifically, the T/FDSA 0120–2026 standard establishes excellent chemistry, manufacturing, and controls (CMC) criteria for quality and safety—including specifications for particle size, zeta potential, residual solvent levels, and microbial limits. However, it stops short of defining therapeutic dosing windows or posology guidelines. The standard does not address what constitutes an effective dose versus a toxic dose for any specific indication. Thus, while the CMC framework is a necessary prerequisite, the pharmacological dosing question remains entirely open. This gap reinforces the critical urgency of the standardization pathways proposed in this perspective. Similarly, the lack of standardized yield reporting has not yet been elevated to a core topic within the existing standardization frameworks.

## 6 Potential pathways toward dose standardization

**FIGURE 1:**
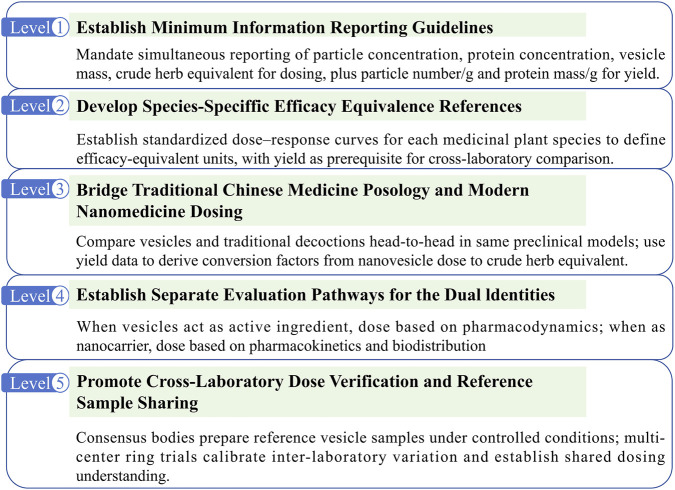
Schematic illustration of the proposed pathways toward dose standardization of TCM-derived extracellular vesicles.

Based on the preceding analysis, we propose several pathways for advancing dose standardization of TCM-derived vesicles, as summarized in Figure 1.

First, establish multi-dimensional reporting norms for dose information and yield data. The experience of the MISEV guidelines in the mammalian EVs field demonstrates that clear reporting checklists significantly aid field-wide standardization (Thieron et al., 2024). TCM vesicle research could adopt a similar approach. Specifically, any publication involving therapeutic applications should report doses in at least four dimensions: particle concentration (particles/mL or particles/kg), protein concentration (mg/mL or mg/kg), vesicle mass concentration (mg/mL or mg/kg), and lipid concentration (nmol phospholipid/mL or mg lipid/kg). Lipid quantification is particularly critical because protein assays are highly susceptible to confounding by abundant co-isolated plant proteins—Rubisco, for instance, can constitute up to 50% of soluble protein in leaf-derived vesicle preparations (Matsushima and Matsuo, 2024). A protein-based dose may therefore primarily reflect contaminating non-vesicular protein rather than actual vesicle quantity. Lipid bilayer quantification provides a much more accurate reflection of the true vesicular dose administered to the subject. We therefore recommend that all PDNV dosing studies report at least one lipid-based metric alongside protein and particle data. Yield data must also be included. We recommend reporting both particle count per Gram of raw herb and protein mass per Gram of raw herb. This provides a basis for cross-study conversion. The honeysuckle vesicle study provided both *in vivo* dose (30 mg/kg/d) and *in vitro* range (50–200 μg/mL) (Gong et al., 2025). The ginseng vesicle study reported both *in vivo* (10 μg/mouse) and *in vitro* (10 μg/mL) data (Zhu et al., 2025). These serve as existing reference models. Muhammad et al. reported lyophilized yields for ginger, garlic, and turmeric side-by-side in one paper (Muhammad et al., 2025). This is a helpful example of standardized yield reporting. If such reporting becomes a community norm, subsequent meta-analyses and dose-response modeling will have a solid data foundation.

Second, establish species-specific efficacy equivalent references. Vesicles from different plant sources possess distinct chemical compositions, biological activities, and extraction yields. A single universal conversion formula is unrealistic and unnecessary. A more pragmatic approach is to establish standardized dose-response curves for each specific medicinal plant. Using one or more stable pharmacodynamic readouts as a reference, researchers can define an efficacy equivalent unit for that plant’s vesicles. The establishment of efficacy equivalents depends on yield data. Only by understanding the baseline yield of a specific herb under a standard protocol can meaningful potency comparisons be made across laboratories.

Third, bridge the relationship between traditional TCM dosage and modern nanomedicine dosage. TCM-derived vesicles are nano-scale components extracted from herbs with centuries of clinical use. Their dosage should not be completely disconnected from traditional empirical knowledge. Future studies should compare vesicles and traditional decoctions head-to-head in the same disease model. Researchers should use yield data to back-calculate nanovesicle doses into the mass of raw herb required to produce them under a standardized, non-denaturing extraction protocol. This will clarify the quantitative relationship between the two forms. However, a critical caveat must be explicitly stated: traditional decoctions are prepared by boiling herbs in water for extended periods. Under these conditions, native phospholipid bilayer-enclosed PDNVs are completely destroyed due to thermal degradation of lipids and denaturation of associated proteins. The nanoparticles that remain in decoctions—or that form *de novo* during heating—are primarily Maillard reaction products and lipid self-assemblies that lack the characteristic EV surface markers, miRNA cargo, and active uptake mechanisms of native PDNVs (Ke et al., 2015). Therefore, comparing mechanically extracted fresh-juice PDNVs to thermally processed decoctions involves comparing ontologically distinct entities. Direct mathematical equivalencies—e.g., “X mg of PDNV protein corresponds to Y grams of raw herb in decoction”—are biologically unsound unless the decoction is prepared under non-denaturing conditions, which is not standard TCM practice. The field must acknowledge this fundamental disjunction; the only valid comparison is between PDNVs and decoctions as separate therapeutic modalities, not as dose-equivalent forms. Such work will not only advance dose standardization. It will also anchor TCM vesicle research more securely within the theoretical and practical foundations of traditional Chinese medicine.

Fourth, design distinct evaluation strategies for the two roles of vesicles. If the research focuses on the intrinsic pharmacological activity of vesicles, dosing should be determined by dose-response relationships based on pharmacodynamic endpoints. Dose-finding approaches common in natural product drug discovery are appropriate here. If the research utilizes vesicles as nanocarriers, dose design must follow nanomedicine principles. Dosing should be guided by pharmacokinetic data and biodistribution studies. If a single study involves both functions, the paper should explicitly clarify the dosing rationale for each role and justify the choices made.

Fifth, promote cross-laboratory dose comparisons and reference sample sharing. The EV field has accumulated experience in this area. Initiatives like the EV-TRACK database and ring trials organized by the International Society for Extracellular Vesicles demonstrate the utility of multi-center collaborations for calibrating experimental variation. The TCM vesicle field can follow a similar path. Established consensus bodies could take the lead in preparing reference vesicle samples from specific herbs under strictly controlled conditions. Multiple laboratories could then use these identical reference samples to generate dose-response curves. This would allow for calibration of systematic inter-laboratory biases. It would also foster a shared understanding of adequate and effective dosing levels. Cross-laboratory calibration of yield data is an inseparable component of this process.

A crucial question is how this proposed framework would interface with existing database infrastructure such as EV-TRACK. EV-TRACK, a community-driven knowledgebase, systematically captures experimental metadata to enhance reproducibility of EV studies. The TCM vesicle field could extend this model by adding EV-TRACK modules specifically for TCM-specific metadata fields: botanical authentication identifiers (linking to MPNS or POWO records, as demonstrated in Section 2), Dao-di origin information, harvest season, post-harvest processing methods, and the specific parts of the medicinal plant used. Such an extension would enable systematic tracking of how these TCM-specific variables affect vesicle yield and bioactivity—directly addressing the dose incomparability problem identified in Table 1. We encourage the EV-TRACK consortium and the Chinese Herbal Medicine Vesicle Expert Committee to explore this integration pathway.

## 7 Conclusion

Research on TCM-derived extracellular vesicles stands at a critical juncture. Exploratory findings continue to emerge rapidly. Yet, the mechanistic elucidation and clinical translation phases demand higher methodological rigor. The issue of dose standardization may appear to be a narrow technical detail. In truth, it directly impacts the scientific credibility and translational trustworthiness of the field. The absence of standardized yield data further fundamentally constrains progress toward dose standardization. A therapeutic modality for which one cannot precisely define how much is extracted from a given amount of herb, nor how much is enough to administer, will struggle to traverse the path from laboratory bench to clinical bedside. This is a particularly salient point for an endeavor that seeks to integrate the ancient wisdom of TCM with the precision tools of twenty-first-century nanomedicine. The multi-dimensional dose reporting framework and yield standardization recommendations proposed in this article are a response to and an elaboration of this urgent call. Only by constructing rigorous metrological bridges between tradition and modernity can TCM-derived extracellular vesicles complete their transformation from laboratory stars into viable clinical drug candidates.

The path forward requires not only scientific consensus but also regulatory action. We call upon national regulatory bodies—including the National Medical Products Administration (NMPA) of China and the U.S. Food and Drug Administration (FDA)—to establish cross-disciplinary working groups that include experts in TCM pharmacognosy, extracellular vesicle biology, nanomedicine, and metrology. These working groups should develop specific guidance documents for PDNV dose reporting, reference material certification, and posology for first-in-human trials. Without such regulatory engagement, the standardization pathways proposed here will remain theoretical exercises. We urge the research community to prioritize this translational agenda.

## Data Availability

The original contributions presented in the study are included in the article/supplementary material, further inquiries can be directed to the corresponding author.
